# Advanced photon counting CT imaging pipeline for cardiac phenotyping of apolipoprotein E mouse models

**DOI:** 10.1371/journal.pone.0291733

**Published:** 2023-10-05

**Authors:** Alex J. Allphin, Ali Mahzarnia, Darin P. Clark, Yi Qi, Zay Y. Han, Prajwal Bhandari, Ketan B. Ghaghada, Alexandra Badea, Cristian T. Badea

**Affiliations:** 1 Quantitative Imaging and Analysis Lab, Department of Radiology, Duke University Medical Center, Durham, NC, United States of America; 2 Department of Radiology, Baylor College of Medicine, Houston, Texas, United States of America; 3 Department of Radiology, Texas Children’s Hospital, Houston, Texas, United States of America; 4 Department of Neurology, Duke University Medical Center, Durham, NC, United States of America; University of Pisa, ITALY

## Abstract

**Background:**

Cardiovascular disease (CVD) is associated with the apolipoprotein E (APOE) gene and lipid metabolism. This study aimed to develop an imaging-based pipeline to comprehensively assess cardiac structure and function in mouse models expressing different APOE genotypes using photon-counting computed tomography (PCCT).

**Methods:**

123 mice grouped based on APOE genotype (APOE2, APOE3, APOE4, APOE knockout (KO)), gender, human NOS2 factor, and diet (control or high fat) were used in this study. The pipeline included PCCT imaging on a custom-built system with contrast-enhanced in vivo imaging and intrinsic cardiac gating, spectral and temporal iterative reconstruction, spectral decomposition, and deep learning cardiac segmentation. Statistical analysis evaluated genotype, diet, sex, and body weight effects on cardiac measurements.

**Results:**

Our results showed that PCCT offered high quality imaging with reduced noise. Material decomposition enabled separation of calcified plaques from iodine enhanced blood in APOE KO mice. Deep learning-based segmentation showed good performance with Dice scores of 0.91 for CT-based segmentation and 0.89 for iodine map-based segmentation. Genotype-specific differences were observed in left ventricular volumes, heart rate, stroke volume, ejection fraction, and cardiac index. Statistically significant differences were found between control and high fat diets for APOE2 and APOE4 genotypes in heart rate and stroke volume. Sex and weight were also significant predictors of cardiac measurements. The inclusion of the human NOS2 gene modulated these effects.

**Conclusions:**

This study demonstrates the potential of PCCT in assessing cardiac structure and function in mouse models of CVD which can help in understanding the interplay between genetic factors, diet, and cardiovascular health.

## Introduction

Aging, sedentary lifestyles, and diets rich in fats and sugars pose significant health concerns due to their association with cardiovascular disease (CVD) and Alzheimer’s disease (AD) [1–3]. Non-invasive *in vivo* imaging methods such as X-ray computed tomography (CT) allow for longitudinal *in vivo* monitoring that is crucial to understanding these diseases [4], particularly, in this case, CVD. Advanced techniques like dual-energy (DE) CT can provide spectral information crucial for atherosclerotic plaque imaging [5] or myocardial perfusion [6]. Yet, the progress of such technology is hindered by the constraints of energy-integrating detectors. A promising alternative lies in using photon-counting detectors (PCD) for photon-counting computed tomography (PCCT), with the potential to considerably enhance CT contrast and enable quantitative material separation with just a single CT scan [7].

Our group has developed preclinical PCCT technology and demonstrated its value in cancer [8] and cardiac studies in mice [9]. Despite these advances, PCCT’s potential for cardiac imaging remains underexplored. To address this gap, we developed a comprehensive preclinical cardiac PCCT imaging pipeline for phenotyping CVD in mice. The pipeline covers several key components: in vivo cardiac PCCT imaging, multi-energy cardiac reconstruction, material decomposition, 3D cardiac segmentation, and quantitative analysis of cardiac function and anatomy. We focus here on using PCCT to study different Apolipoprotein E (APOE) genotypes in mice. APOE is a key protein involved in the metabolism of lipids in the body. The roles of the three major human APOE alleles, specifically APOE2, APOE3, APOE4, and APOE-/- (i.e., APOE Knock Out (KO)) are of particular interest due to their distinct association with CVD [2]. APOE2 is linked to type III hyperlipoproteinemia, while APOE4 is associated with elevated low density lipoprotein (LDL) levels and a higher risk of atherosclerosis [2]. While the effects of APOE isoforms on lipid metabolism and CVD in mice are generally similar to humans, there are also some differences. Mice primarily have higher levels of high density lipoprotein (HDL) levels, whereas humans have higher levels of LDL [10].

Our study encompasses both male and female mice carrying the three human APOE alleles and fed either a normal, control diet (CTRL) or a high-fat diet (HFD), to model the diet-related risks that APOE4 carriers face compared to APOE3 controls [11, 12]. We also incorporate mouse models with a humanized innate immune system, i.e., the inclusion of the human (h)NOS2 gene (presence is indicated by the inclusion of the acronym HN, e.g., APOE2HN). In the context of CVD, the role of NOS2 and its product nitric oxide is complex. While nitric oxide has protective roles in the cardiovascular system, such as vasodilation and inhibition of platelet aggregation, overproduction of nitric oxide by NOS2 in pathological conditions can lead to oxidative stress, promoting inflammation and atherosclerosis [13, 14]. However, human NOS2 specifically hasn’t been studied in APOE mouse models.

Alternative imaging methods have been used in mice for cardiac studies. For example, both echocardiography [15, 16] and MRI [17] have been used to examine the cardiac and vascular characteristics of APOE KO mice. In the absence of APOE, mice develop severe atherosclerosis due to the accumulation of remnant lipoproteins, thereby underlining the crucial role of APOE in lipoprotein metabolism and CVD [18, 19]. However, echocardiography lacks the anatomical detail of CT imaging, while MRI, despite its high-resolution capabilities, tends to be more expensive and does not typically provide isotropic spatial resolution. Moreover, the calcified plaques in APOE KO mice are best imaged using CT.

## Materials and methods

Our phenotyping pipeline included contrast enhanced *in vivo* PCCT imaging, retrospective intrinsic cardiac gating, spectral and temporal iterative reconstruction, spectral decomposition, and deep learning cardiac segmentation. Left ventricle segmentations were then used to statistically evaluate the effects of genotype, diet, sex, and body weight on cardiac function and anatomy.

### Mouse models

All mice were bred and maintained in facilities managed by Duke University Medical Center in the Bryan Research Building for Neurobiology, and all animal procedures were approved by the Duke IACUC (protocol registry number: A173-20-08). Our study included a total of 123 mice. The mice were divided into 28 groups depending on their genotype, gender, presence of HN factor, and diet (CTRL or HFD) (see Table 1). Table 2 provides a simplified summary of the composition within these groups using percentages of sex, HN, and diet for each APOE isoform. For mice on HFD, we have used the D12451 diet (Research Diet, New Brunswick, NJ) starting at ~9 months of age for a duration of 12 weeks (see Table 3). For reference, we also present the control diet (LabDiet, Rodent Diet 5001).

**Table 1:** Counts for each experimental group when grouped by diet, genotype (differentiating HN factor), and sex.

Diet	Genotype	Sex	Group Count
CTRL	APOE2	Male	6
HFD	APOE2	Female	4
HFD	APOE2	Male	5
CTRL	APOE2HN	Female	3
CTRL	APOE2HN	Male	3
HFD	APOE2HN	Female	3
HFD	APOE2HN	Male	3
CTRL	APOE3	Female	5
CTRL	APOE3	Male	3
HFD	APOE3	Female	4
HFD	APOE3	Male	10
CTRL	APOE3HN	Female	3
CTRL	APOE3HN	Male	3
HFD	APOE3HN	Female	7
HFD	APOE3HN	Male	7
CTRL	APOE4	Female	3
CTRL	APOE4	Male	3
HFD	APOE4	Female	2
HFD	APOE4	Male	7
CTRL	APOE4HN	Female	4
CTRL	APOE4HN	Male	7
HFD	APOE4HN	Female	5
HFD	APOE4HN	Male	4
CTRL	APOE KO	Female	4
CTRL	APOE KO	Male	5
HFD	APOE KO	Female	6
HFD	APOE KO	Male	4

**Table 2:** Overview of experimental group composition with percentages of HN and HFD in each genotype.

Genotype	Male	Female	%HN	%HFD
APOE2	17	10	44.4%	55.6%
APOE3	23	19	47.6%	66.7%
APOE4	21	14	57.1%	51.4%
APOE KO	9	10	N/A	52.6%

This table has been included primarily to communicate the relative counts for each experimental factor (i.e., sex, HN factor, and diet).

**Table 3:** Nutritional details of the diets referred to as HFD and CTRL.

	D12451 (HFD)	LabDiet5001 (CTRL)
**Protein (kcal%)**	20	28.9
**Fat (kcal%)**	45	13.6
**Carbohydrate (kcal%)**	35	57.5
**Energy Density (kcal/g)**	4.73	2.89
**Sucrose (%)**	17	3.25
**Cholesterol(mg/Kg)**	201	196

### *In vivo* PCCT imaging

We performed all micro-CT cardiac imaging using our own custom PCCT system [20]. The system is equipped with a Varian G297 x-ray tube and a Santis 1604 PCD (Dectris, Inc). The PCD has 4 energy bins, 150 μm pixels and a 1 mm CdTe sensor. The field of view of the system is approximately 12.9 cm x 4.3 cm.

The animals were anesthetized during scanning using 2–3% isoflurane delivered by nosecone in our custom animal cradle. Breathing was monitored using a pneumatic pillow with a pressure transducer, and ECG was monitored using electrodes placed on the mouse paws and foot. The mice were administered liposomal iodine (Lip-I) via retro-orbital injection, with a dosage set at 0.012 ml/g of body weight. The injection process typically spanned a duration of 10 to 20 seconds reducing potential discomfort for the animals. After the injection, the mice were monitored for 5 minutes before imaging to ensure there were no adverse reactions. A heat lamp was used to maintain a healthy, comfortable temperature for each mouse. At the end of the study, the mice were euthanized using an intraperitoneal injection of 250 mg/Kg pentobarbital, as approved by our institution’s animal care and use committee. We ensured that all actions were carried out humanely, and with the utmost regard for the welfare of our animal subjects. Lip-I contrast agent was fabricated similar to methods described previously [21, 22]. The I concentration in the Lip-I formulation is 110 ± 4 mg/ml. The average liposome size was 145 ± 6 nm. We have previously reported on using these types of liposomal nanoparticles for vascular imaging using spectral PCCT imaging [8].

**Fig 1:**
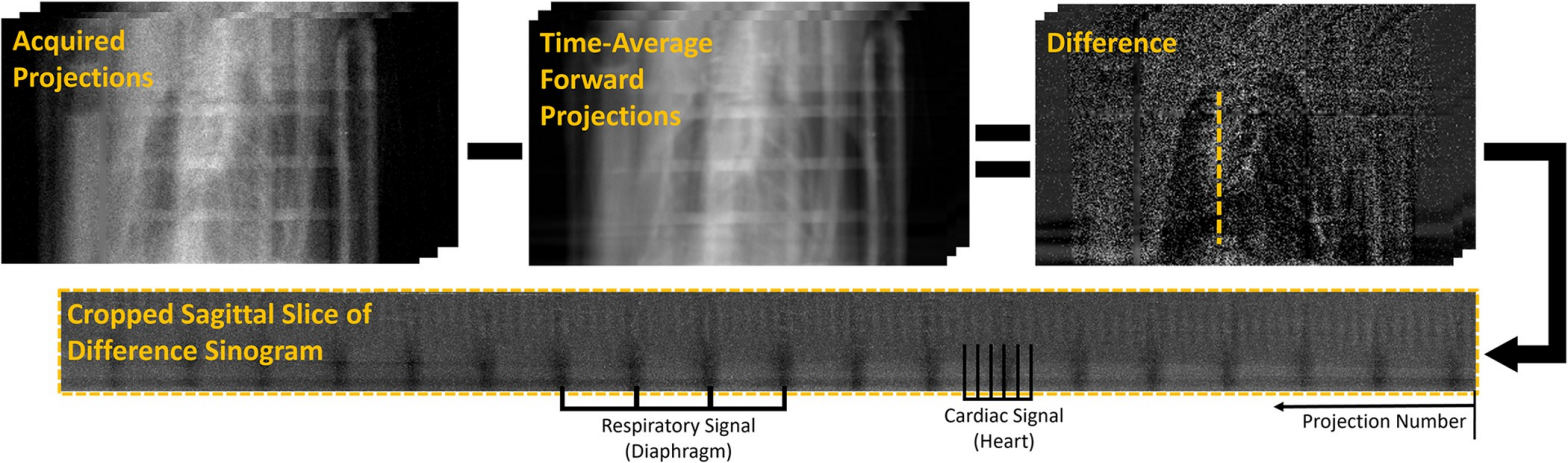
Overview of our retrospective, intrinsic gating approach. We acquire a full set of ungated projections. We then reconstruct the full data set with no gating and forward project this volume to get a set of time-average projections. We then subtract the time-average forward projections from the original projections. This difference sinogram contains those features from the original sinogram which vary with time (i.e., respiration and cardiac motion). For this work, we have only used the cardiac signal.

Next, a PCCT scan was executed with the following parameters: 80 kVp tube voltage, 4 mA tube current, 10 ms/exposure. 7000 views were captured over a total scan time of 70 seconds. The absorbed radiation dose associated with imaging was ~118 mGy. The PCD thresholds were set to 25, 34, 50, and 60 keV. These thresholds were chosen specifically to benefit the K-edge of iodine (33.2 keV) and thus to maximize the contrast enhancement. The projections corresponding to 10 different cardiac phases were sorted and identified through a semi-automated, projection-domain intrinsic gating procedure. Fig 1 illustrates the intrinsic gating procedure which allows for identification of R-R intervals and calculation of heart rate directly from the projection data. This procedure, in essence, utilizes the difference between calculated forward projections of an ungated reconstruction and the acquired projections to obtain a sinogram signal almost exclusively containing features showing cardiac and respiratory motion. Performing a Fourier transform of this sinogram and filtering frequencies outside of the range of expected heart rates (300–600 bpm), we isolate the cardiac signal. This analysis is valid if the sampling rate is sufficiently fast to avoid aliasing. In our case, a sampling rate of 100 Hz (10 ms/exposure) was deemed satisfactory. Intrinsic gating has the inherent benefit of having no need for high fidelity measurement of ECG signals because cardiac information is extracted directly from the acquired projection data. Though we did use a basic ECG measurement to verify subject health during scanning, no ECG signal was used for image gating.

### 5D CT reconstruction

The PCCT data were jointly reconstructed using a multi-channel iterative algorithm and an isotropic voxel size of 125 microns. Specifically, we applied the split Bregman method with the add-residual-back strategy [23] and both 4D bilateral filtration (4DBF) [24, 25] and rank-sparse kernel regression (RSKR) [26] regularization. Reconstruction iteratively computed *X* that is the solution to the following equation:

$$\hat{X}=\underset{X}{\arg\;\min}\frac{1}{2}\sum_{t}\sum_{e}\left[{\left||RX(t,e)-Y(t,e)|\right|}_{2}^{2}+\lambda (t,e)Reg(X(t,e))\right]$$ (1)

The reconstructed data (X) at each energy (e) and time (t) minimizes the reprojection error (R representing the system projection matrix) relative to log-transformed projection data (Y). Projections are temporally selected based on the intrinsic gating procedure previously discussed. To reduce noise in the reconstructed results, this data fidelity term is minimized subject to penalizing the bilateral total variation (BTV) term. 4DBF is used as the regularizer (Reg) along the time dimension while RSKR minimizes BTV within and between energies.

Reconstruction resulted in 5D volumes with 4 energies, 10 cardiac phases, and 3 spatial dimensions with 125-μm isotropic voxels. Iterative reconstruction times ranged from 5–9 hours depending on the volume size.

### Material decomposition

The inherent spectral capabilities of the PCD allowed us to perform material decompositions of these volumes using a single scan. We performed image-based material decomposition on PCD iterative reconstructions using the method of Alvarez and Macovski [27] extended to include iodine as a K-edge material. Each cardiac volume was decomposed into material maps corresponding to iodine (I), the photoelectric effect (PE), and Compton scattering (CS) [9]. We also decomposed a phantom, which will be described in the next section, into I, calcium (Ca), and H_2_O material maps for the specific purpose of quantifying Ca sensitivity. Material vials of known concentrations in each scan allowed for proper calibration of the material decomposition sensitivity matrix. Material decomposition was performed by matrix inversion, solving the following linear system at each voxel:

$$C=XM^{‐1}.$$ (2)

In this formulation, X is the reconstructed PCCT images, C represents the concentrations of our basis materials (e.g., I, PE and CS or I, Ca, and H_2_O) for each voxel, and M is a matrix of material sensitivities at each energy. An orthogonal subspace projection approach was used to prevent negative concentrations [26]. Post-decomposition, the material maps were assigned color maps and combined in ImageJ for visualization.

### Calcified plaque detectability

To assess the limit of detectability of calcified plaques, especially critical for APOE KO mice, we designed a custom phantom, composed of cylinders of varying diameters filled with three distinct concentrations of Ca and one concentration of I arranged in a grid. The cylinder diameters ranged from 0.5 mm to 2.5 mm. Ca concentrations were 10 mg/mL, 20 mg/mL and 40 mg/mL. The I concentration was 10 mg/ml. We calculated the contrast-to-noise ratio (CNR), as defined in Eq 3, for each combination of concentration and diameter.

$$CNR=\frac{\mu_{signal}‐\mu_{background}}{\sigma_{background}}$$ (3)

The primary signal of interest in these calculations was Ca, set against an I background, simulating the conditions encountered in contrast-enhanced CT scans where differentiating calcified plaques from iodine-enhanced blood is critical. CNR values were calculated for both the lowest energy threshold PCD image and the Ca map from an I-Ca-H2O material decomposition. When using the PCD images, the signal was the attenuation measured in the Ca vial while the background was the attenuation measured in the I value. We note that larger attenuation values in the I vial compared to the Ca vials can lead to negative CNR values indicating an inability to detect Ca within an I background. When using the decomposed Ca images, the signal was the concentration of Ca in the calcium vial while the background was the concentration of Ca in the iodine vial.

### Cardiac segmentation

To quantitatively assess cardiac function, we segmented the left ventricle of each mouse in both the diastolic and systolic phases. User-guided 3D segmentations were first performed using ITK-Snap (http://www.itksnap.org/), specifically the snake-based region growing tool. After performing the comparatively tedious and repetitive segmentations using ITK-Snap, we used the resulting data to train a convolutional neural network (CNN) to eliminate the need for user-guided segmentation within this pipeline. There was a total of 246 cardiac segmentations performed with the user-guided approach. These segmentations included the systolic and diastolic phases from all mice reported in Table 1. 186 segmentations were used for training, 38 for validation, and 22 were set aside for testing. All data, independent of cardiac phase or mouse, were randomly shuffled prior to group separation. To improve generalizability and avoid overfitting, data augmentation strategies including random shifts in image intensity, random zooming, and random rotations were used. The network architecture for the CNN was a U-Net with 3D convolution operations similar to [28]. The input and output of the network were 3D volumes (128^3^ voxels). Images were normalized such that the mean and the standard deviations were 0 and 1 respectively.

To determine the most effective input to our deep learning segmentation, we trained two different CNNs with the same structure, one using the lowest energy CT image as the network input and another one using the decomposed iodine map as the input. We chose to do this to explore the potential benefit of using spectral data for segmentation tasks. In both cases, the labels used for network training and evaluation were the ITK-Snap user-assisted segmentations performed on the lowest threshold PCCT images.

Our network was trained using the sum of binary cross entropy and 1 minus the Dice coefficient:

$${Loss}_{Total}={Loss}_{BCE}+{Loss}_{Dice}$$ (4)

Eq 5 shows the binary cross entropy portion of the loss function. In this equation, *x_i_* represents the ground truth segmentation (0 or 1) and *y_i_* represents the predicted segmentation probabilities (between 0 and 1).

$${Loss}_{BCE}=-\frac{1}{N}\sum_{i=1}^{N}x_{i}∙\log\left(y_{i}\right)+(1-x_{i})∙\log\left(1-y_{i}\right)$$ (5)

Eq 6 shows the definition of the Dice coefficient portion of the loss function. In this equation, *X_i_* represents the ground truth segmentation map and *Y_i_* represents a binarized version of the predicted segmentation map in which probabilities greater than or equal to 0.5 have been set to 1 and values less than 0.5 have been set to 0. |*X_i_*| and |*Y_i_*| represent the cardinalities of the two maps. Thus, the numerator of Eq 6 equates to 2 times the number of true positive predictions; and the denominator equates to the total number of positively valued voxels within both the predicted and actual segmentation maps.

$${Loss}_{Dice}=-\frac{1}{N}\sum_{i=1}^{N}1-\frac{2|X_{i}\cap Y_{i}|}{\left|X_{i}|+|Y_{i}\right|}$$ (6)

CNN training and deployment were implemented using the PyTorch framework. We trained for 50 epochs using the Adam optimizer with a learning rate of 5e-4. The learning rate decreased by a factor of 0.1 if the validation loss plateaued for longer than 5 epochs.

We report CNN segmentation performance using Dice score, precision, recall, and area under the receiver operating characteristic curve (AUC). In order to determine an optimal decision threshold, we used the threshold that minimized the difference between precision and recall for the training data. Using that threshold, we calculated these evaluation metrics using the 22 test segmentations. We report these metrics as averages accompanying associated standard deviations.

### Statistical analysis

Statistical results were obtained following linear model analyses using the R programming language. Our analysis compared the effect and interaction in mice of sex (Male or Female), their age, weight or diet (e.g., HFD or CTRL), and their genotype (APOE2, APOE2HN, APOE3, APOE3HN, APOE4, APOE4HN, or KO) on a variety of cardiac measurements. Our measurements included the diastolic left ventricular volume (DLVV), systolic left ventricular volume (SLVV), stroke volume (SV) calculated as the difference between DLVV and SLVV, cardiac output (CO) calculated as SV multiplied by the heart rate (HR), ejection fraction (EF) determined as 100 times SV divided by DLVV, and cardiac index (CI) obtained by dividing CO by the weight of the mouse. The most popular definition of CI is the ratio of CO and the body surface area (BSA); however, due to difficulty to measure the BSA we replaced it with body weight as in [29]. Heart rate was calculated using the intrinsic gating procedure previously described. The heart rate was measured while the mice were under anesthesia. Body weight was measured prior to imaging using a digital scale. All other cardiac measurements were calculated from the CNN-based segmentations of the left ventricle. The correlation between different cardiac measurements was calculated. Given that diet is binary, and body weight is a continuous random variable, we employed the Biserial correlation coefficient to assess the association between diet and body weight. The correlation coefficient is found to be remarkably high (0.756), accompanied by a significantly small p-value of 8e-22. Consequently, we select one of these covariates for our linear model to avoid issues of co-linearity. Body weight was chosen over diet due to its continuous nature and its potential to better capture the residual variation of the model. This choice is justified by the fact that most of our outcome variables are continuous and can be more suitably modeled using a continuous measure.

Our analysis fitted linear models to the data to analyze the relationship between different variables and the cardiac measurements. Analysis of variance (ANOVA) was used to test for significant differences between groups, and post-hoc tests were performed using the R `emmeans`package [https://cran.r-project.org/web/packages/emmeans/index.html]. To ensure a balanced study design with respect to age, we eliminated outliers which are defined as samples with age that deviate beyond 1.5 times the interquartile range (IQR) from the first quartile (Q1) and third quartile (Q3) within each of the eight groups (four genotypes multiplied by two sexes; see Table 2).

We next employed linear models for each cardiac measure e.g., Cardiac output ~ Sex*Age*Weight*HN*Genotype, where Genotype represents APOE2,3,4, and APOE KO. HN is 0 for APOE2, APOE3, and APOE4 mouse lines with mNos2 background, and 1 for APOE2 mNos2^-/-^ hNOS, APOE3 mNos2^-/-^ hNOS, and APOE4 mNos2^-/-^ hNOS. We calculated Cohen F effect sizes, confidence intervals, and adjusted our post hoc results using the Tukey method [30]. The results are presented both numerically and visually to provide a comprehensive understanding of the findings.

## Results

### PCCT cardiac reconstruction

**Fig 2:**
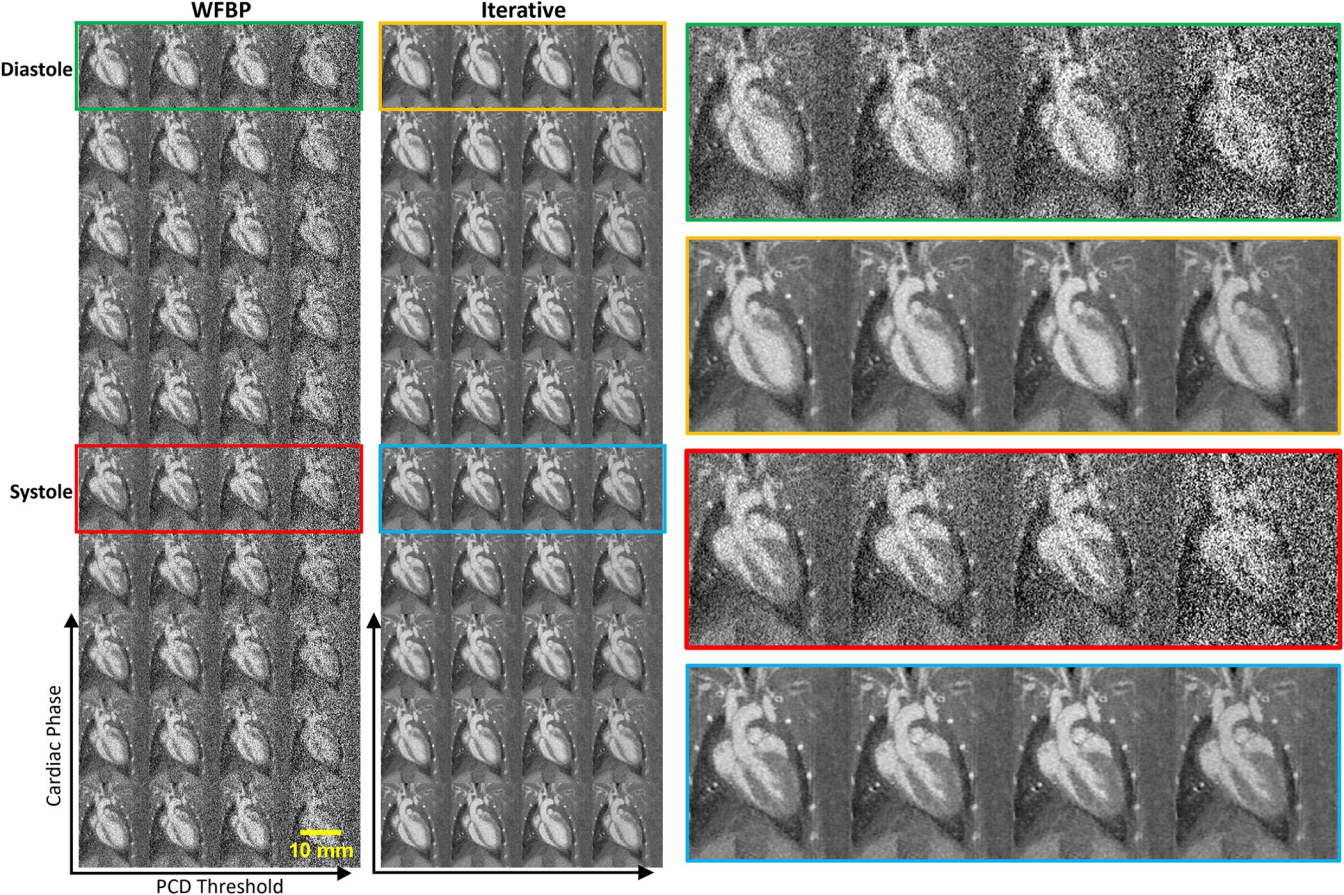
Coronal slices from an example 5D reconstruction of an APOE3 mouse on the HFD. The two large, bounded image columns on the left contain collections of all 4 PCD threshold images sampled at each of the 10 cardiac phases. The left image collection shows images from the WFBP reconstruction. The right collection shows images from the iterative reconstruction. These 40-image collections show the 10 cardiac phases along the Y axis and the 4 PCD energy thresholds along the X axis. In the far right of this figure, we show larger versions of the diastolic and systolic cardiac phase images. Colored image borders indicate where these larger images fit within the full collection shown. All images are windowed from -1000 HU to 1500 HU.

A representative 5D cardiac reconstruction is shown in Fig 2. Both the weighted filtered back projection (WFBP) [31] and the iterative reconstruction are shown for noise level comparison. The difference in noise level is especially clear in the sets reconstructed with weighted filtered backprojection and corresponding to the highest energy threshold (i.e., 60 keV) due to the lower number of photons. However, our multi-channel iterative reconstruction is very effective in reducing noise. The standard deviation in the water vial decreased from approximately 662 HU to 84 HU for this highest energy image when reconstructing iteratively.

### Material decomposition and plaque detection

**Fig 3:**
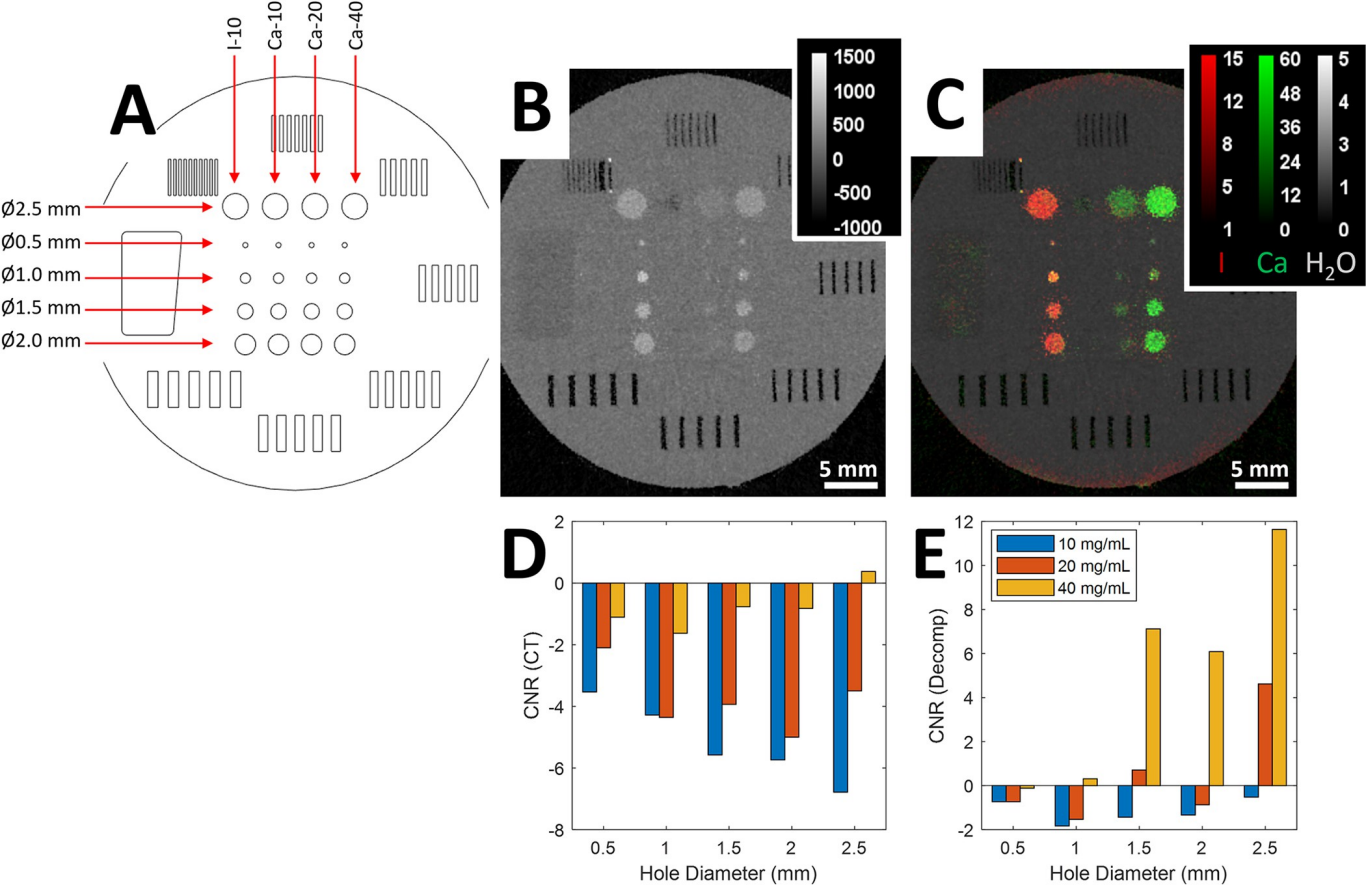
Overview of quantification of calcified plaque detectability. (A) The diagram shows the design of the custom phantom. The phantom consists of cylinders of different diameters which have been filled with different concentrations of Ca or I. (B) A single energy axial CT slice. (C) Composite color representation of the associated I-Ca-H2O decomposition. (D) The plots below the axial slices show the CNR values for single energy CT and (E) for the Ca decomposition. CNR values were calculated according to Eq 3 as described previously.

CNR calculations from our custom phantom (Fig 3) showed that single-energy CT images were unable to detect Ca signal within an I background for all concentrations and diameters tested based on Rose Criterion which requires CNR > 3 [32]. Negative CNR values for single energy CT images suggest the dominance of the I signal, effectively masking any underlying Ca signal. However, in the decomposed Ca maps, we obtained a CNR value of 4 or more for diameters down to 1.5 mm at a 40 mg/mL Ca concentration, and for a diameter down to 2.5 mm at a 20 mg/mL Ca concentration. From these results, we infer that our system can reliably identify plaques as small as 1–1.5 mm. Although this might undervalue the system’s performance given the pure Ca and I environments, it provides a conservative estimate of plaque detectability and starkly illustrates the superiority of spectral CT in this domain.

**Fig 4:**
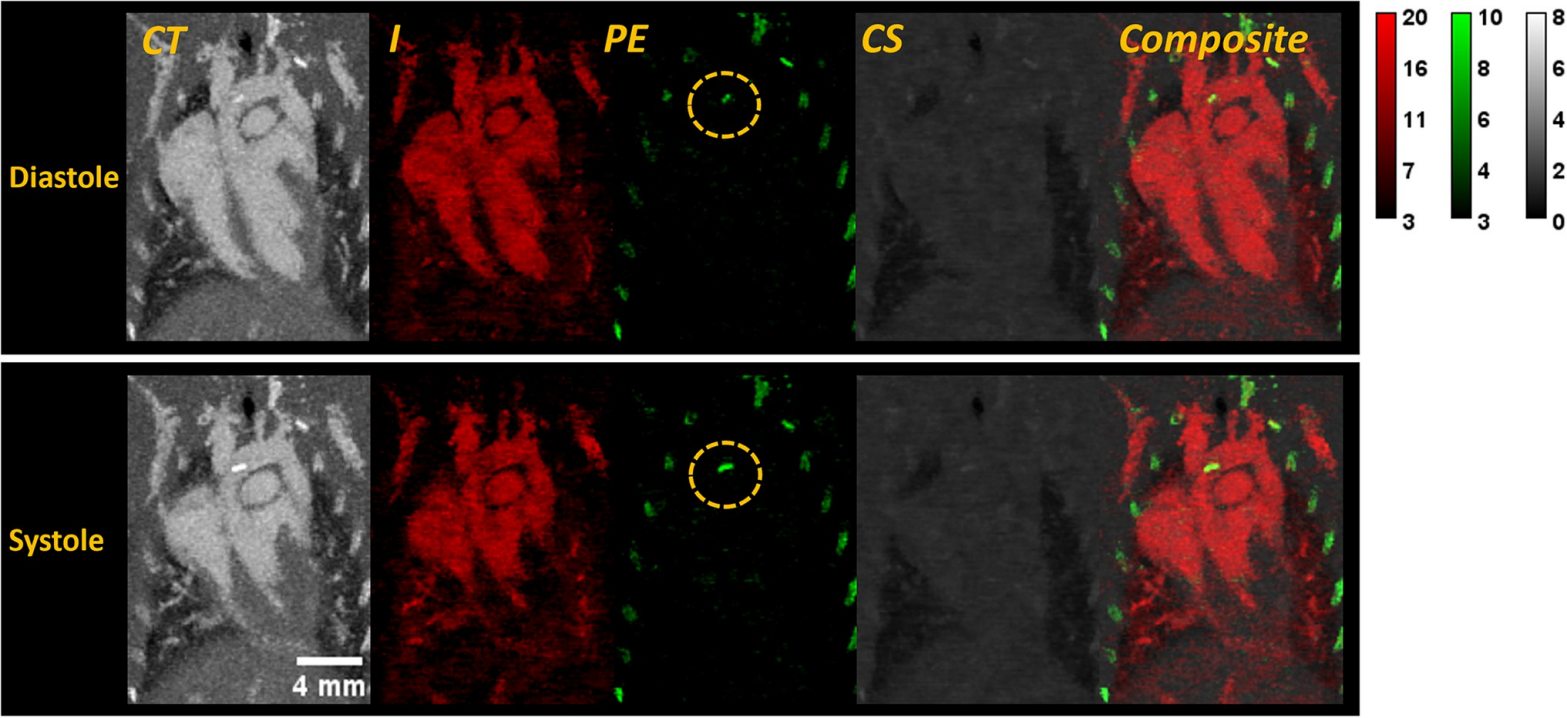
Coronal MIPs of a representative I-PE-CS decomposition from a APOE KO mouse on the HFD. One energy of the corresponding CT images is shown on the far left followed by the I, PE, and CS decomposition maps. The I map is shown in red. The PE map is shown in green. The CS map is shown in gray. The far-right image shows a composite image with all 3 decomposition maps. We show examples from both the diastolic and systolic cardiac phases. The PE map enables clear identification of a calcified aortic plaque. Without spectral capabilities, it can be more difficult to confidently identify these plaques. The CT images are windowed from -500 to 2000 HU. The decomposed material maps are windowed according to the color bars shown. I is given in mg/mL. PE and CS are given as fractions of the attenuation of water.

Fig 4 shows a representative set of spectrally decomposed images of an APOE KO mouse on the HFD. None of the other genotypes had visible atherosclerotic plaques. It is widely recognized that APOE KO mice develop atherosclerotic plaques [33] which are prominently observed in this mouse model along the inferior portion of the aortic arch. It is important to note that the calcified plaques are clearly visible in the photoelectric (PE) maps. Thus, spectral PCCT imaging allows for the straightforward differentiation of these plaques from the blood pool, even when a contrast agent is used.

### Cardiac segmentation

**Fig 5:**
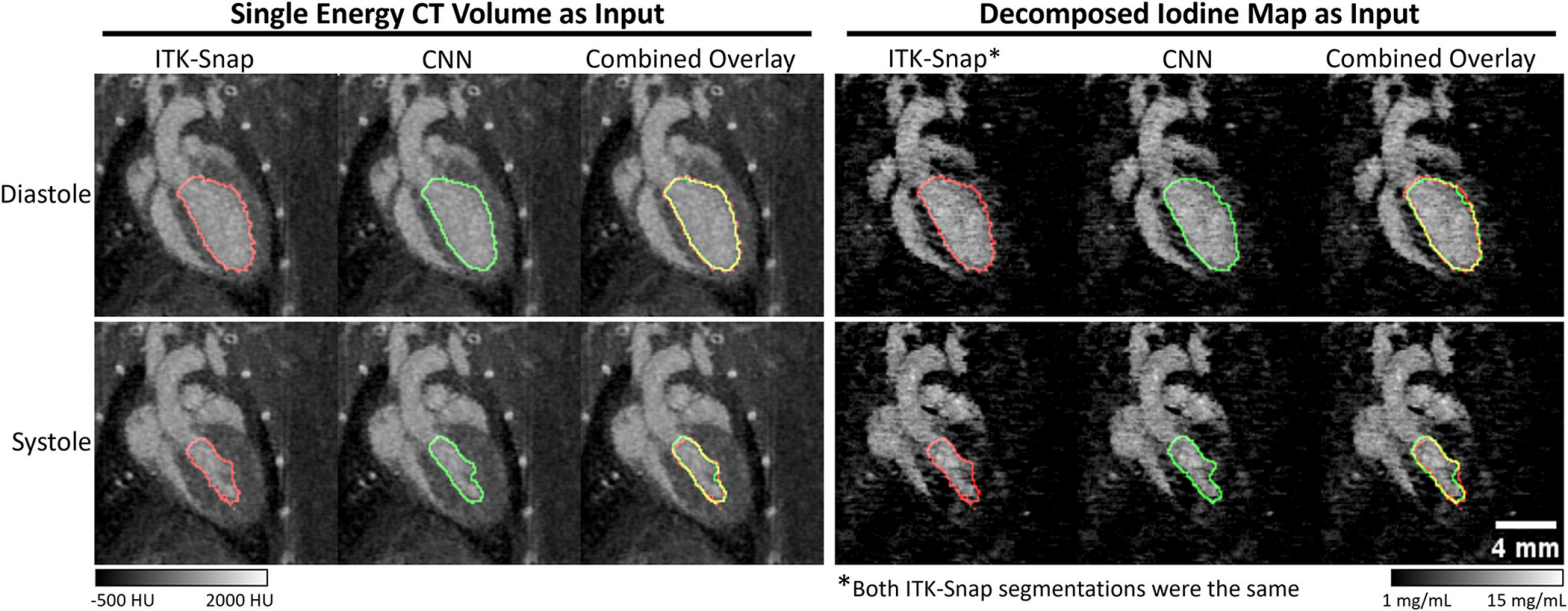
CNN-based left ventricle segmentation results. This figure shows the traced outline of various segmentations within coronal slices of the inputs for the two CNNs. We have included both the diastolic and systolic phases to illustrate the performance across different phases of the heart. The left column shows the results when training using single energy CT volumes while the right column shows the results when training using decomposed iodine maps. We show the user-guided segmentations from ITK-Snap, the predicted CNN outputs, and combined overlays of the two segmentations. Yellow boundaries in the combined images indicate a perfect match between the CNN and label while visible regions of green or red indicate an over or under estimation.

**Fig 6:**
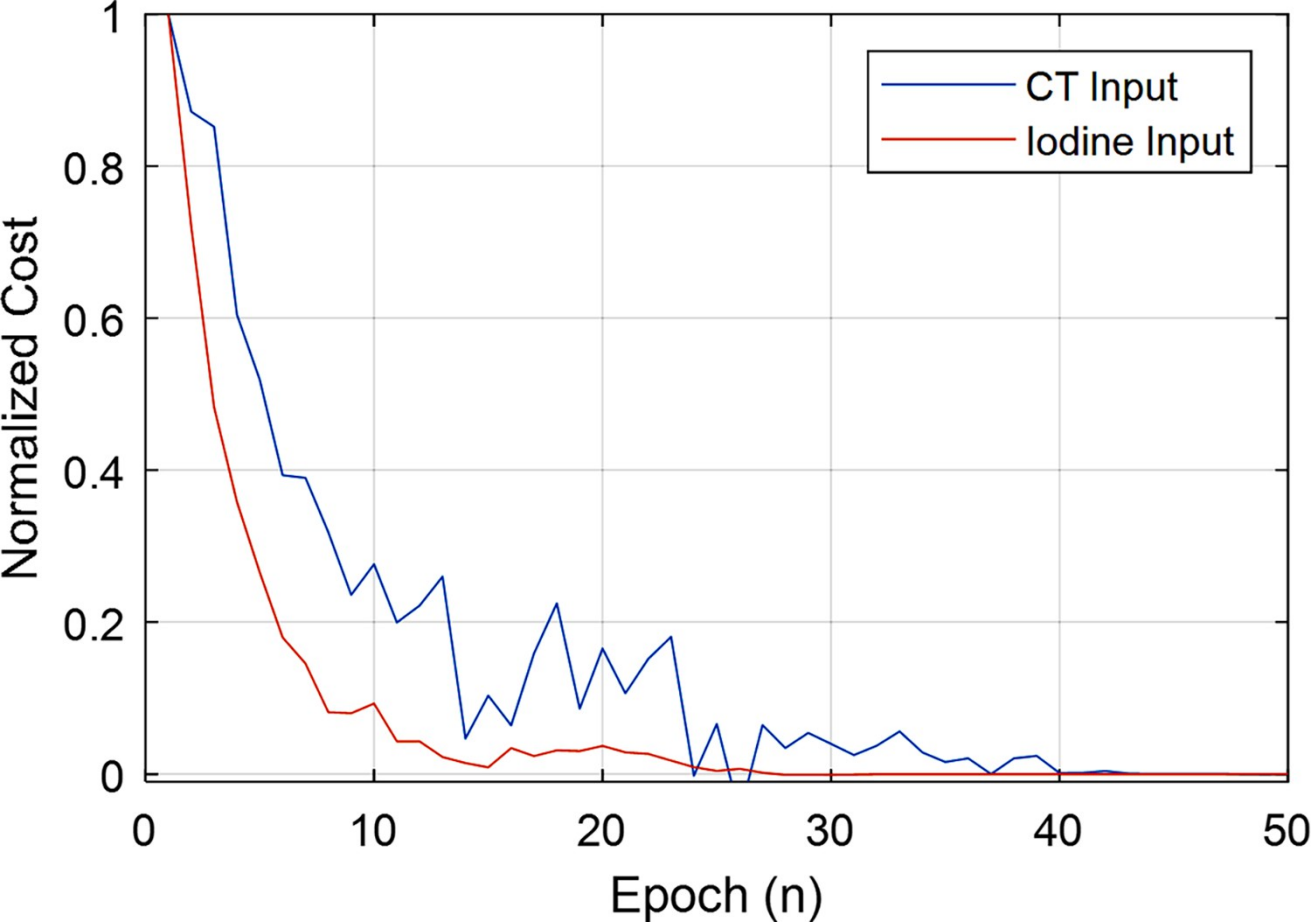
CNN segmentation validation loss throughout training. We show here the validation loss (normalized from 0 to 1) as a function of epoch number throughout CNN training. This is intended to illustrate the difference in convergence between the two network inputs.

Example segmentations from ITK-Snap and the two CNNs are shown in Fig 5. Within the scope of pipeline development, we sought to determine the most accurate choice of CNN. From a qualitative perspective both CNNs perform remarkably well with only minor over or under estimations along the ventricle boundaries. Though not substantiated numerically, there appear to be some regions in which the CNNs more accurately capture the ventricle contours, most notably near the papillary muscle. Table 4 shows the quantitative performance of the CNNs in relation to the ITK-Snap labels. We note that the labels were generated using the CT volumes; separate labels were not created using the iodine maps. From a quantitative perspective, the CT volume CNN slightly outperforms the iodine map CNN with a test Dice score of 0.91 compared to the lower score for the iodine CNN of 0.89. However, as shown in Fig 6, the iodine map CNN has a more stable and rapid convergence. Based on the superior Dice score, we chose to use the CNN trained using CT volumes within our pipeline.

**Table 4:** CNN segmentation performance metrics for training, validation, and test sets.

	Training (n = 186)		Validation (n = 38)		Test (n = 22)	
	CT Input	Iodine Input	CT Input	Iodine Input	CT Input	Iodine Input
Dice	0.90 ± 0.06	0.88 ± 0.06	0.89 ± 0.07	0.87 ± 0.07	0.91 ± 0.03	0.89 ± 0.05
Precision	0.90 ± 0.09	0.88 ± 0.10	0.89 ± 0.11	0.85 ± 0.12	0.94 ± 0.06	0.91 ± 0.06
Recall	0.91 ± 0.09	0.89 ± 0.09	0.91 ± 0.07	0.90 ± 0.07	0.88 ± 0.07	0.88 ± 0.08
AUC	0.96 ± 0.05	0.95 ± 0.04	0.96 ± 0.04	0.96 ± 0.03	0.95 ± 0.03	0.94 ± 0.04

We report the Dice coefficient, precision, recall, and AUC for both the CNN which was trained using single-energy CT volumes as input and the CNN which was trained using decomposed iodine maps as input.

### Statistical analysis

Table 5 summarizes the average and standard deviation of the cardiac metrics grouped by sex, genotype, and diet. The parameters assessed are weight, DLVV, SLLV, HR, SV, EF, CO, and CI. The measures are given as average values with standard deviations in parentheses. The data indicates that male mice on a HFD consistently weigh more than those on the CTRL diet across all genotypes. This pattern is also seen in female mice, suggesting that diet has a significant effect on body weight irrespective of sex and genotype. Male APOE2 mice on HFD show the largest average DLVV and SLVV values among males, while female APOE3 mice on HFD show the largest average values among females. The APOE KO genotype mice exhibit high variation in these parameters as indicated by the large standard deviations. In males, APOE2 mice on CTRL diet show the highest HR, while in females, APOE KO mice on HFD show the highest average heart rate. SV is consistent across all groups, apart from a slight decrease observed in mice on HFD. Mice on CTRL diet tend to have higher ejection fractions than those on HFD, irrespective of sex and genotype. Both CO and CI tend to decrease with HFD in most groups. It’s important to note that the significant variations indicate a high level of individual variability, emphasizing the need for large sample sizes in such studies.

**Table 5:** Average and standard deviation for each quantitative metric grouped according to sex, genotype, and diet.

Sex	Genotype	Diet	Weight [g]	Diastolic LV [mL]	Systolic LV [mL]	HR [bpm]	Stroke Volume [mL]	Ejection Fraction [%]	Cardiac Output [mL/min]	Cardiac Index [mL/min*g]
**Male**	*APOE2*	CTRL	31.678 (1.707)	0.045 (0.004)	0.022 (0.008)	470.857 (48.618)	0.023 (0.008)	51.027 (17.141)	11.065 (5.167)	0.348 (0.158)
		HFD	42.867 (6.491)	0.062 (0.01)	0.038 (0.008)	419.429 (59.411)	0.024 (0.007)	38.075 (8.667)	10.11 (3.582)	0.243 (0.101)
	*APOE3*	CTRL	33.75 (5.381)	0.052 (0.012)	0.031 (0.012)	430.428 (15.451)	0.021 (0.003)	42.218 (12.067)	9.142 (1.584)	0.274 (0.046)
		HFD	48.494 (6.185)	0.048 (0.008)	0.028 (0.008)	442.84 (34.142)	0.02 (0.007)	42.008 (12.651)	8.772 (2.837)	0.183 (0.059)
	*APOE4*	CTRL	25.738 (5.554)	0.036 (0.01)	0.019 (0.007)	444.536 (26.801)	0.017 (0.005)	48.639 (6.871)	7.698 (2.287)	0.307 (0.09)
		HFD	46.462 (6.118)	0.05 (0.01)	0.024 (0.009)	421.179 (45.692)	0.026 (0.006)	53.559 (14.81)	10.895 (2.869)	0.239 (0.071)
	*APOE KO*	CTRL	31 (0.753)	0.058 (0.022)	0.027 (0.03)	465.214 (45.272)	0.031 (0.01)	61.278 (28.481)	14.561 (4.998)	0.47 (0.161)
		HFD	42.75 (4.186)	0.041 (0.012)	0.02 (0.008)	458.143 (19.602)	0.021 (0.014)	49.189 (26.629)	9.568 (6.544)	0.234 (0.166)
**Female**	*APOE2*	CTRL	27.867 (6.768)	0.044 (0.015)	0.015 (0.001)	466.857 (20.217)	0.03 (0.015)	64.758 (9.64)	13.871 (7.295)	0.481 (0.147)
		HFD	36.96 (9.162)	0.044 (0.005)	0.02 (0.007)	424.629 (37.291)	0.025 (0.005)	55.961 (12.243)	10.567 (2.814)	0.287 (0.046)
	*APOE3*	CTRL	29.438 (1.586)	0.037 (0.006)	0.021 (0.007)	448.821 (28.004)	0.017 (0.006)	45.116 (17.153)	7.535 (3.074)	0.259 (0.11)
		HFD	49.927 (7.298)	0.049 (0.012)	0.023 (0.012)	443.688 (32.04)	0.026 (0.008)	54.671 (16.885)	11.492 (3.845)	0.237 (0.093)
	*APOE4*	CTRL	28.4 (2.772)	0.038 (0.009)	0.019 (0.008)	455.755 (51.694)	0.018 (0.009)	48.459 (21.048)	8.648 (4.824)	0.305 (0.169)
		HFD	45.971 (8.331)	0.044 (0.011)	0.019 (0.009)	421.469 (53.904)	0.024 (0.006)	57.232 (13.29)	10.113 (2.399)	0.222 (0.049)
	*APOE KO*	CTRL	26.15 (2.428)	0.035 (0.006)	0.018 (0.003)	457.071 (27.163)	0.017 (0.008)	46.26 (16.109)	7.822 (3.869)	0.306 (0.172)
		HFD	31.175 (8.095)	0.039 (0.01)	0.021 (0.013)	486.643 (47.267)	0.018 (0.002)	49.308 (17.073)	8.807 (2.017)	0.3 (0.106)

**Fig 7:**
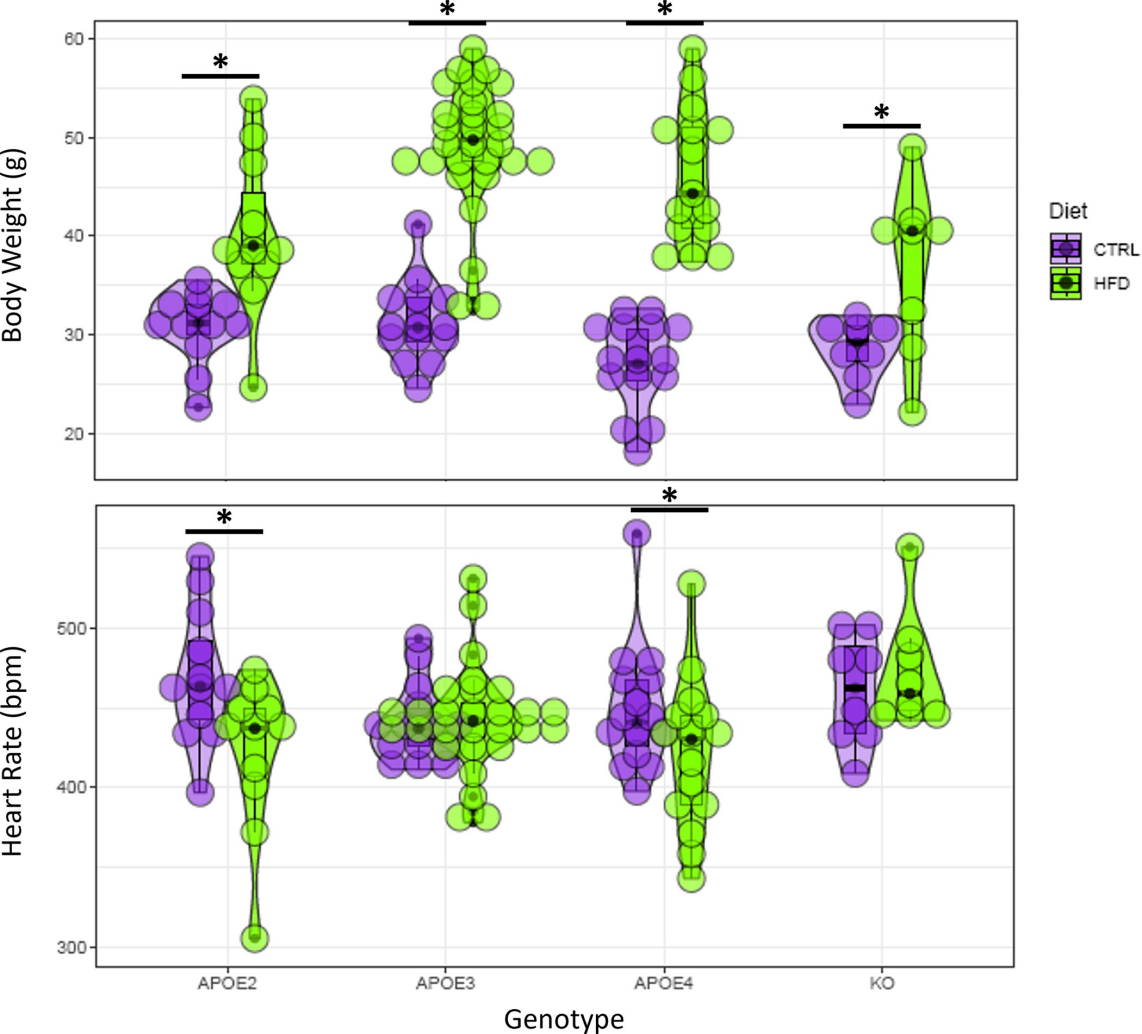
Violin plots of two key imaging agnostic metrics: Body weight and heart rate. The X axis corresponds to the 4 genotype groups: APOE2, APOE3, APOE4, and APOE KO. Diet groups (CTRL and HFD) within each genotype are color coded. Asterisks indicate significant comparisons (p<0.05).

**Fig 8:**
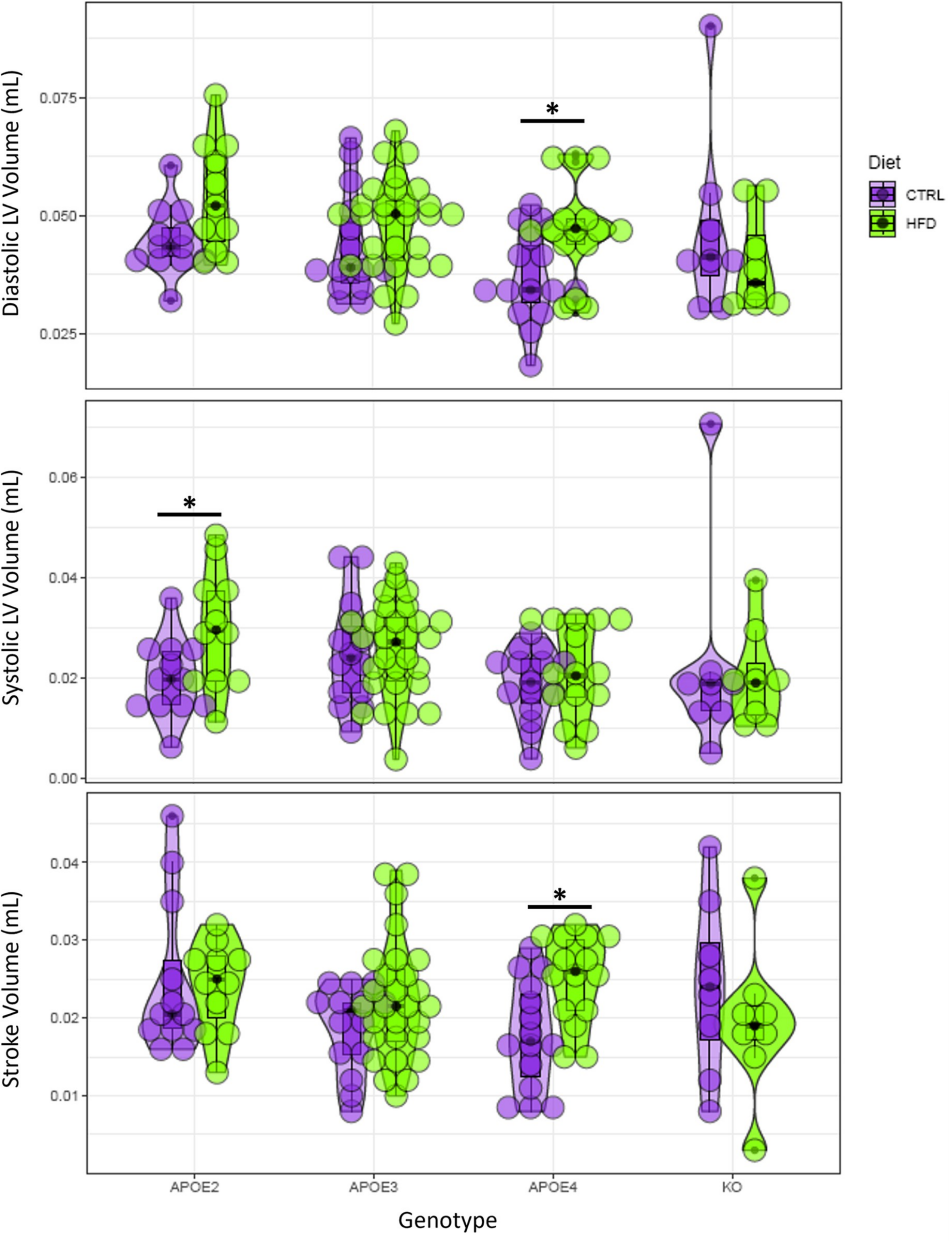
Violin plots of three image-derived cardiac metrics: Diastolic left ventricle volume, systolic left ventricle volume, and stroke volume. The X axis corresponds to the 4 genotype groups: APOE2, APOE3, APOE4, and APOE KO. Diet groups (CTRL and HFD) within each genotype are color coded. Asterisks indicate significant differences (p<0.05).

**Fig 9:**
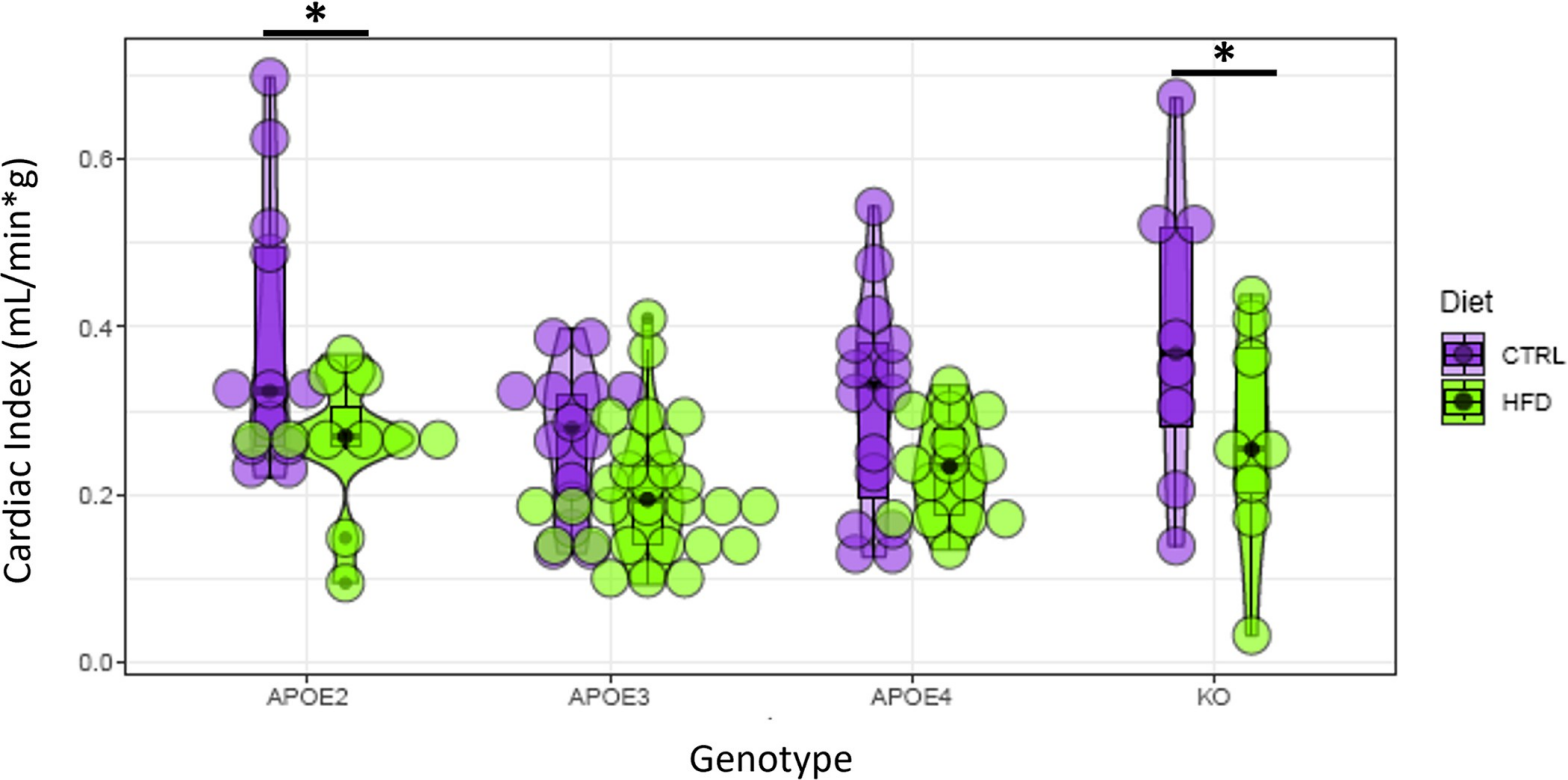
Violin plots of cardiac index. The X axis corresponds to the 4 genotype groups: APOE2, APOE3, APOE4, and APOE KO. Diet groups (CTRL and HFD) within each genotype are color coded. Asterisks indicate significant differences (p<0.05).

Our statistical analysis revealed a variety of significant factors and significant group differences summarized in the following plots and tables. Figs 7–9 present a visual overview using violin plots of some key quantitative statistical results. These plots group the data based on genotype (APOE2, APOE3, APOE4, and APOE KO) and show CTRL and HFD as different colors. These figures specifically highlight differences within each genotype between CTRL or HFD mice. For example, Fig 7 shows that all genotypes showed a significant increase in body weight on the HFD.

More interestingly, the results of our analysis reveal that APOE genotype influences various cardiac measurements. For example, genotype was found to be a significant predictor of DLVV, HR, SV, CO, and CI. Additionally, there were significant differences between the CTRL and HFD diets for the APOE2 and APOE4 genotypes for HR and SV. These results suggest a difference in how diet affects the cardiac health of different genotypes.

Sex and body weight were found to be significant predictors of several cardiac measurements, including DLVV, SLVV, and CI. This suggests these factors may also play a role in determining cardiac health.

**Fig 10:**
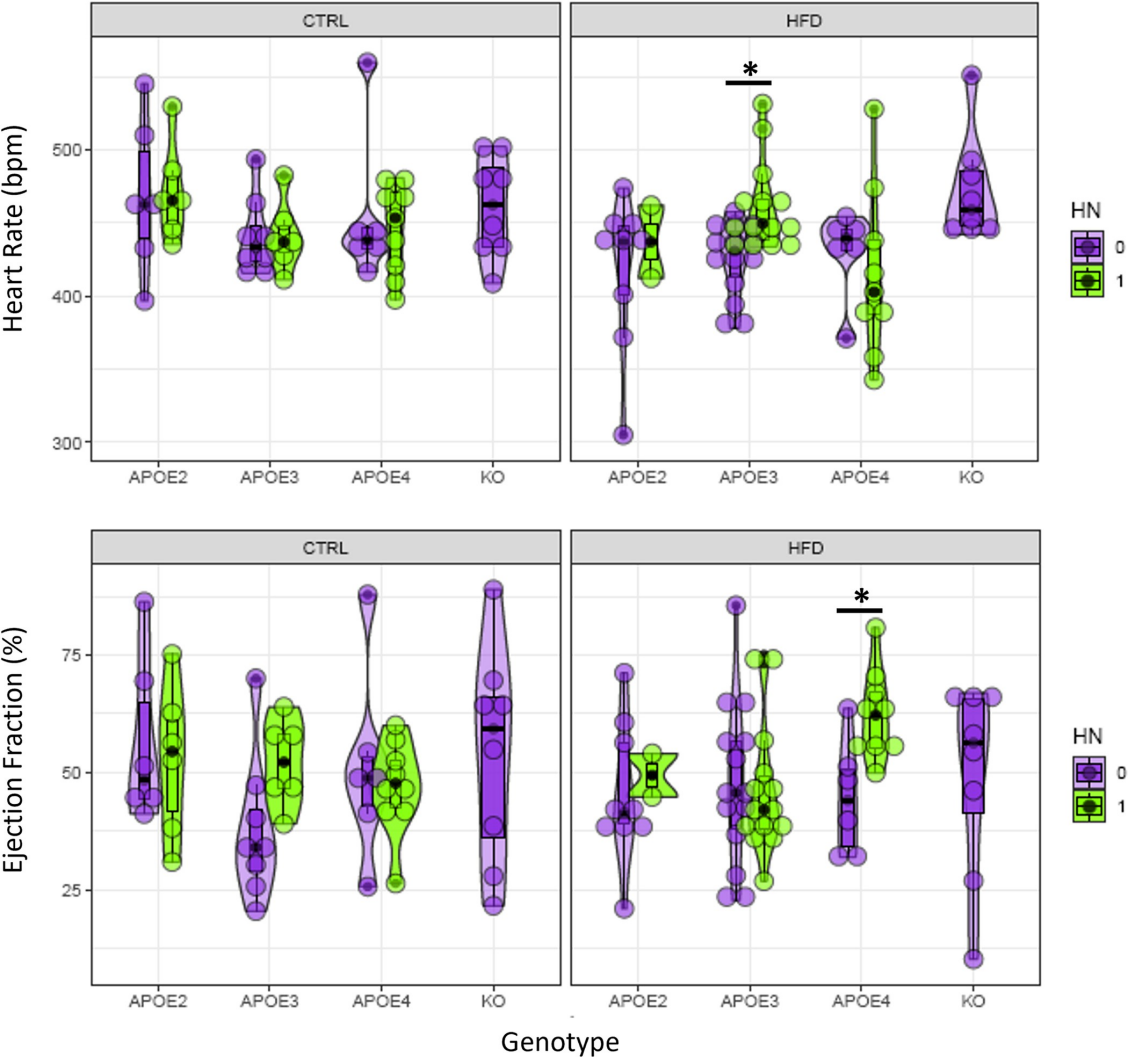
Violin plots of diastolic HR and ejection fraction. The X axis corresponds to the 4 genotype groups: APOE2, APOE3, APOE4, and APOE KO. Diet groups (CTRL and HFD) are separated into left and right bounding boxes. The presence (1) or absence (0) of the human HN factor is indicated by violin color. Asterisks indicate significant differences (p<0.05).

Fig 10 provides an overview of significant differences stemming from the inclusion of the human NOS2 gene (HN) that renders the mouse immune response more human like. HN was found to have a significant effect on HR for the APOE3 genotype on the HFD as well as on ejection fraction for the APOE4 genotype on the HFD.

Table 6 summarizes the findings regarding which factors have significant effects on cardiac metrics. Age had a significant effect on Diastolic LV Volume, while Sex and body weight were significant factors for both the systolic and diastolic LV volume. Weight was also important for the cardiac index. There was a significant interaction of weight by genotype for ejection fraction, cardiac output, and cardiac index. HN was important for the stroke volume changes in APOE4 mice on HFD, and for heart rate in APOE3 mice on HFD. Heart rate showed significant differences with diet in APOE2 and APOE4 mice. Stroke volume was different between APOE4 mice on CTRL and HFD diet, while cardiac index was different between APOE2 mice on CTRL and HFD diet, and between APOE KO mice in CTRL and HFD. Our results illustrate the complex interplay between APOE genotype, age, sex, and diet in modulating cardiac function, as well as the increased sensitivity of APOE2 and APOE4 human alleles compared to APOE3 allele.

**Table 6:** Complete table of significant factors and group differences including P-values and a basic interpretation.

Variable	Predictor Or Comparison	p-value	Interpretation
Diastolic LV Volume	Sex	0.003595	Sex is a significant predictor of diastolic LV volume.
Diastolic LV Volume	Age	0.013701	Age is a significant predictor of diastolic LV volume.
Diastolic LV Volume	Weight	0.00093	Weight is a significant predictor of diastolic LV volume.
Diastolic LV Volume	Genotype	0.035696	APOE genotype is a significant predictor of diastolic LV volume.
Diastolic LV Volume	CTRL—HFD (APOE4)	0.013755	There is a significant difference in diastolic LV volume between the CTRL and HFD diets for the APOE4 genotype.
Systolic LV Volume	Sex	0.003549	Sex is a significant predictor of systolic LV volume.
Systolic LV Volume	Weight	0.022497	Weight is a significant predictor of systolic LV volume.
Systolic LV Volume	CTRL—HFD (APOE2)	0.042098	There is a significant difference in systolic LV volume between the CTRL and HFD diets for the APOE2 genotype.
Heart Rate	Genotype	0.025583	APOE genotype is a significant predictor of heart rate.
Heart Rate	CTRL—HFD (APOE2)	0.001657	There is a significant difference in heart rate between the CTRL and HFD diets for the APOE2 genotype.
Heart Rate	CTRL–HFD (APOE4)	0.039345	There is a significant difference in heart rate between the CTRL and HFD diets for the APOE4 genotype.
Heart Rate	HN0—HN1 (APOE3, HFD)	0.032348	There is a significant difference in heart rate between HN0 and HN1 for the APOE3 genotype on the HFD diet.
Stroke Volume	Genotype	0.043067	There is a significant difference in stroke volume between the different levels of APOE genotype (APOE2, APOE3, APOE4 and APOE KO).
Stroke Volume	CTRL—HFD (APOE4)	0.011397	There is a significant difference between CTRL and HFD diets for the APOE4 genotype, with HFD diet having a higher mean stroke volume than CTRL diet.
Ejection Fraction	Weight:Genotype	0.002788	There is a significant difference in ejection fraction between the different levels of APOE genotype (APOE2, APOE3, APOE4 and APOE KO) that depends on weight.
Ejection Fraction	HN0—HN1 (APOE4, HFD)	0.04825	There is a significant difference in ejection fraction between HN0 and HN1 for the APOE4 genotype on the HFD diet.
Cardiac Output	Genotype	0.023867	There is a significant difference in cardiac output between the different levels of APOE genotype (APOE2, APOE3, APOE4 and APOE KO).
Cardiac Output	Weight:Genotype	0.031181	There is a significant difference in cardiac output between the different levels of APOE genotype (APOE2, APOE3, APOE4 and APOE KO) that depends on weight.
Cardiac Index	Weight	2.06E-07	There is a significant difference in cardiac index between the different levels of weight.
Cardiac Index	Genotype	0.021818	There is a significant difference in cardiac index between the different levels of APOE genotype (APOE2, APOE3, APOE4 and APOE KO).
Cardiac Index	Weight:Genotype	0.038939	There is a significant difference in cardiac index between the different levels of APOE genotype (APOE2, APOE3, APOE4 and APOE KO) that depends on weight.
Cardiac Index	CTRL—HFD (APOE2)	0.010592	There is a significant difference between CTRL and HFD diets for the APOE2 genotype, with HFD diet having a significantly lower cardiac index than CTRL diet.
Cardiac Index	CTRL—HFD (APOE KO)	0.045401	There is a significant difference between CTRL and HFD diets for the APOE KO genotype, with HFD diet having a significantly lower cardiac index than CTRL diet.

We have only included figures and tables which include at least one statistically significant finding. Our online repository contains all relevant results.

## Discussion and conclusion

We have demonstrated the capabilities of preclinical PCCT to effectively estimate conventional cardiac metrics. Compared to echocardiography, PCCT offers enhanced 3D visualization, reduced noise levels, and additional spectral information that can benefit in separation of atherosclerotic plaques. Compared to MRI, PCCT is faster and provides isotropic resolution which can give higher accuracy in segmentation of the heart chambers and therefore better accuracy in assessing cardiac metrics. CT imaging involves the use of ionizing radiation, but in our studies, the radiation dose (~118 mGy) represents 55 to 76 times less than the lethal dose of 6.5–9 Gy, known as LD50/30 [34]. Thus, we do not consider this a major limitation, and repeated imaging sessions to study the disease longitudinally, are feasible.

We note the importance of achieving properly cardiac gated, low-noise iterative reconstructions within our imaging pipeline. At its core, the pipeline’s effectiveness is dependent on CT reconstruction quality. Blurry ventricle boundaries, due to noise or improper gating, will directly impact segmentation quality. All our image-based cardiac metrics are derived from these segmentations. To achieve quantitative reliability and repeatability, high-quality reconstruction is paramount.

We also note the key benefits and promise of CNN-based segmentation. CNN segmentations are fast, and provide reduced user bias [28]. In a user-guided segmentation task, some subjectivity is involved when identifying boundaries such as the boundary between the aorta and the left ventricle. CNNs learn to recognize patterns across a full dataset eliminating bias that may be present for a single user-guided segmentation instance.

In this work, we explored the possible benefit of using decomposed material maps for segmentation. Training speed and stability were superior for the CNN trained using iodine maps. The CNN trained using CT images showed superior quantitative performance in terms of Dice coefficient. It is likely that the superior performance of our CNN trained using CT images came because the labels themselves were created using CT images. Future work will involve exploring additional benefits of using decomposed material maps for biological segmentation tasks.

Overall, our results provide valuable insights into the complex interplay between APOE genotype, diet, sex, weight, and HN in determining cardiac health. We specifically revealed key differences in how the APOE genotypes respond to HFD. APOE2 and APOE4 genotypes are known to be high risk factors for AD and CVD [2]. In our study, the APOE4 genotype showed significant differences in HR and SV between the control and HFD. The APOE2 genotype also exhibited significant differences in HR and CI when comparing the two diets. This suggests that these genotypes may be more susceptible to diet-induced changes in cardiac function, highlighting a potential genetic predisposition. The inclusion of the human NOS2 gene appears to further modulate these effects. The presence of this gene significantly affected HR in the APOE3 genotype and EF in the APOE4 genotype, both on a high-fat diet. This indicates that the presence of the human NOS2 gene could amplify the impact of a high-fat diet on cardiac function in these genotypes. Such information may help in more complex studies of the interplay between human NOS2 gene and its effect on CVD.

Sex and body weight were also found to significantly influence various cardiac measures across genotypes, highlighting the role of these factors in cardiovascular health. For example, body weight was a significant predictor of DLVV, SLVV and CI, underscoring the known link between body weight and cardiovascular health.

Taken together, these results illustrate a complex interplay between genotype, diet, sex, and body weight in determining cardiovascular health. It underscores the potential for individual genetic makeup, combined with dietary habits, to influence heart function and health. Recent studies have highlighted that APOE genotypes differentially modulate the association between cardiac output and cognition in older adults [35]. This supports the need for and importance of considering genetic factors in dietary recommendations and the development of interventions for cardiovascular health. The findings could also open the door for more personalized dietary guidelines based on a person’s genetic makeup to optimize heart health and help maintain cognition in older adults.

We note, however, that extrapolating these findings to humans presents certain challenges. Murine and human APOE differ in their amino acid sequences, which can influence protein function and metabolic pathways. Additionally, humans and mice vary in their lipoprotein profiles, with mice having the majority of cholesterol in high-density lipoprotein (HDL) particles, as opposed to LDL particles in humans [10]. Thus, our findings in mice need to be carefully tested also in humans.

In addition to these points, we acknowledge that our PCCT system, while offering superior image quality and material differentiation, is a research tool with limited availability. The application of this technology in a clinical setting requires further validation studies with clinical-grade equipment. Nevertheless, our results are a promising step forward, providing valuable insights into the potential of PCCT and CNN-based image analysis for *in vivo* cardiovascular research.

## References

[1] Mahley R.W. and Rall S.C. Jr, Apolipoprotein E: Far more than a lipid transport protein. Annual Review of Genomics and Human Genetics, 2000. 1(2000): p. 507–537. doi: 10.1146/annurev.genom.1.1.507 11701639

[2] Mahley R.W., Apolipoprotein E: from cardiovascular disease to neurodegenerative disorders. J Mol Med (Berl), 2016. 94(7): p. 739–46. doi: 10.1007/s00109-016-1427-y 27277824 PMC4921111

[3] Liu C.C., et al., Apolipoprotein e and Alzheimer disease: Risk, mechanisms and therapy. Nature Reviews Neurology, 2013. 9(2): p. 106–118. doi: 10.1038/nrneurol.2012.263 23296339 PMC3726719

[4] De Bruijn R.F. and Ikram M.A., Cardiovascular risk factors and future risk of Alzheimer’s disease. BMC Medicine, 2014. 12(1). doi: 10.1186/s12916-014-0130-5 25385322 PMC4226863

[5] Danad I., Ó Hartaigh B., and Min J.K., Dual-energy computed tomography for detection of coronary artery disease. Expert review of cardiovascular therapy, 2015. 13(12): p. 1345–1356. doi: 10.1586/14779072.2015.1102055 26549789 PMC4797067

[6] Jin K.N., et al., Myocardial perfusion imaging with dual energy CT. Eur J Radiol, 2016. 85(10): p. 1914–1921. doi: 10.1016/j.ejrad.2016.06.023 27427412

[7] Taguchi K. and Iwanczyk J.S., Vision 20/20: Single photon counting x-ray detectors in medical imaging. Med Phys, 2013. 40(10): p. 100901. doi: 10.1118/1.4820371 24089889 PMC3786515

[8] Badea C., et al., Functional imaging of tumor vasculature using iodine and gadolinium-based nanoparticle contrast agents: a comparison of spectral micro-CT using energy integrating and photon counting detectors. Physics in Medicine & Biology, 2019. 64(6): p. 065007. doi: 10.1088/1361-6560/ab03e2 30708357 PMC6607440

[9] Clark D.P., et al., Photon-counting cine-cardiac CT in the mouse. PLoS One, 2019. 14(9): p. e0218417. doi: 10.1371/journal.pone.0218417 31536493 PMC6752874

[10] Getz G.S. and Reardon C.A., Animal models of atherosclerosis. Arterioscler Thromb Vasc Biol, 2012. 32(5): p. 1104–15. doi: 10.1161/ATVBAHA.111.237693 22383700 PMC3331926

[11] Martins I.J., et al., Apolipoprotein E, cholesterol metabolism, diabetes, and the convergence of risk factors for Alzheimer’s disease and cardiovascular disease. Mol Psychiatry, 2006. 11(8): p. 721–36. doi: 10.1038/sj.mp.4001854 16786033

[12] Strittmatter W.J., et al., Apolipoprotein E: high-avidity binding to beta-amyloid and increased frequency of type 4 allele in late-onset familial Alzheimer disease. Proc Natl Acad Sci U S A, 1993. 90(5): p. 1977–81. doi: 10.1073/pnas.90.5.1977 8446617 PMC46003

[13] Pautz A., et al., Regulation of the expression of inducible nitric oxide synthase. Nitric Oxide, 2010. 23(2): p. 75–93. doi: 10.1016/j.niox.2010.04.007 20438856

[14] Besedina A., NO-Synthase Activity in Patients with Coronary Heart Disease Associated with Hypertension of Different Age Groups. J Med Biochem, 2016. 35(1): p. 43–49. doi: 10.1515/jomb-2015-0008 28356863 PMC5346800

[15] Vasquez E.C., et al., Cardiac and vascular phenotypes in the apolipoprotein E-deficient mouse. J Biomed Sci, 2012. 19(1): p. 22. doi: 10.1186/1423-0127-19-22 22330242 PMC3306747

[16] Liehn E.A., et al., Heart function assessment during aging in apolipoprotein E knock-out mice. Discoveries (Craiova), 2021. 9(3): p. e136. doi: 10.15190/d.2021.15 34816004 PMC8605688

[17] Li Y., et al., In vivo MRI detection of atherosclerosis in ApoE-deficient mice by using tenascin-C-targeted USPIO. Acta Radiol, 2018. 59(12): p. 1431–1437. 29566551 10.1177/0284185118762613

[18] Piedrahita J.A., et al., Generation of mice carrying a mutant apolipoprotein E gene inactivated by gene targeting in embryonic stem cells. Proc Natl Acad Sci U S A, 1992. 89(10): p. 4471–5. doi: 10.1073/pnas.89.10.4471 1584779 PMC49104

[19] Zhang S.H., et al., Spontaneous hypercholesterolemia and arterial lesions in mice lacking apolipoprotein E. Science, 1992. 258(5081): p. 468–71. doi: 10.1126/science.1411543 1411543

[20] Holbrook M.D., Clark D.P., and Badea C.T., Dual source hybrid spectral micro-CT using an energy-integrating and a photon-counting detector. Phys Med Biol, 2020. 65(20): p. 205012. doi: 10.1088/1361-6560/aba8b2 32702686 PMC7770809

[21] Mukundan S., et al., A Nanoscale, Liposomal Contrast Agent for Preclincal MicroCT Imaging of the Mouse. AJR, 2006. 186: p. 300–307. 16423931 10.2214/AJR.05.0523

[22] Ghaghada K.B., et al., Early Detection of Aortic Degeneration in a Mouse Model of Sporadic Aortic Aneurysm and Dissection Using Nanoparticle Contrast-Enhanced Computed Tomography. Arterioscler Thromb Vasc Biol, 2021. 41(4): p. 1534–1548. doi: 10.1161/ATVBAHA.120.315210 33535789 PMC7990703

[23] Gao H., et al., Multi-energy CT based on a prior rank, intensity and sparsity model (PRISM). Inverse Probl, 2011. 27(11): p. 115012. doi: 10.1088/0266-5611/27/11/115012 22223929 PMC3249839

[24] Clark D.P., et al., Spectrotemporal CT data acquisition and reconstruction at low dose. Med Phys, 2015. 42(11): p. 6317–36. doi: 10.1118/1.4931407 26520724 PMC4600087

[25] Tomasi, C. and R. Manduchi. Bilateral filtering for gray and color images. in Sixth international conference on computer vision (IEEE Cat. No. 98CH36271). 1998. IEEE.

[26] Clark D.P. and Badea C.T., Hybrid spectral CT reconstruction. PLOS ONE, 2017. 12(7): p. e0180324. doi: 10.1371/journal.pone.0180324 28683124 PMC5500339

[27] Alvarez R.E. and Macovski A., Energy-selective reconstructions in X-ray computerized tomography. Phys Med Biol, 1976. 21(5): p. 733–44. doi: 10.1088/0031-9155/21/5/002 967922

[28] Holbrook M.D., et al., MRI-Based Deep Learning Segmentation and Radiomics of Sarcoma in Mice. Tomography, 2020. 6(1): p. 23–33. doi: 10.18383/j.tom.2019.00021 32280747 PMC7138523

[29] Kreissl M.C., et al., Noninvasive measurement of cardiovascular function in mice with high-temporal-resolution small-animal PET. J Nucl Med, 2006. 47(6): p. 974–80. 16741307 PMC4348007

[30] Tukey J.W., Comparing Individual Means in the Analysis of Variance. Biometrics, 1949. 5(2): p. 99–114. 18151955

[31] Karl S., et al., Weighted FBP—a simple approximate 3D FBP algorithm for multislice spiral CT with good dose usage for arbitrary pitch. Physics in Medicine & Biology, 2004. 49(11): p. 2209. doi: 10.1088/0031-9155/49/11/007 15248573

[32] Rose A., The Sensitivity Performance of the Human Eye on an Absolute Scale*. Journal of the Optical Society of America, 1948. 38(2): p. 196–208. doi: 10.1364/josa.38.000196 18901781

[33] Meir K.S. and Leitersdorf E., Atherosclerosis in the Apolipoprotein E–Deficient Mouse. Arteriosclerosis, Thrombosis, and Vascular Biology, 2004. 24(6): p. 1006–1014. 15087308 10.1161/01.ATV.0000128849.12617.f4

[34] Williams J.P., et al., Animal models for medical countermeasures to radiation exposure. Radiat Res, 2010. 173(4): p. 557–78. doi: 10.1667/RR1880.1 20334528 PMC3021126

[35] Bown C.W., et al., Apolipoprotein E Genotype Modifies the Association Between Cardiac Output and Cognition in Older Adults. Journal of the American Heart Association, 2019. 8(15): p. e011146. doi: 10.1161/JAHA.118.011146 31364446 PMC6761646

